# A systematic analysis of in-source fragments in LC-MS metabolomics

**DOI:** 10.1101/2025.02.04.636472

**Published:** 2025-02-05

**Authors:** Yuanye Chi, Joshua M. Mitchell, Shuzhao Li

**Affiliations:** 1 The Jackson Laboratory for Genomic Medicine, 10 Discovery Drive, Farmington, CT 06032, USA; 2 University of Connecticut School of Medicine, Farmington, CT 06032, USA

The “dark matter” of metabolomics refers to the large number of unidentified features in metabolomic studies, mostly from mass spectrometry (MS) based analysis (deSilva2015; David2021; Giera2024). The topic is pertinent to the analytical coverage of small molecules in biomedical research (Kind2009; Uppal2016), approaches to metabolite annotation (Domingo2018; Chaleckis2019; Metz2025), mapping reaction pathways (Zamboni2015) and the promise of applying metabolomics and exposomics to precision medicine (Wishart2016; Vermeulen2020). The number of unidentified features is not a direct account of number of compounds, as a metabolite can have isotopologues, adducts and fragments that are measured in the same data (Mahieu2017; Wang2019; Li2023a). Giera et al (2024) recently reported that in-source fragments (ISFs) accounted for over 70% of MS/MS features in METLIN, one of the leading spectral databases, suggesting that ISFs could be a significant portion of the “dark matter”. Since the reference spectra in METLIN are based on chemical standards, we examine here the LC-MS (liquid chromatography coupled mass spectrometry) metabolomics from biological samples, which are the most relevant in biomedical investigations.

LC-MS metabolomics data from 45 studies were retrieved from public repositories (orbital mass analyzer, electron spray ionization (ESI) in positive or negative mode, Table S1). For consistence of data analysis, only studies of human plasma or serum samples are included, and large datasets are down selected to about 100 samples, because low-frequency features cumulate by sample numbers. We use the asari software (Li2023b) for processing raw data to features of unique m/z (mass-to-charge ratio) and retention time (RT). Asari is designed with explicit data models that report all features above a quality threshold (signal-to-noise ratio, or SNR, of 2, peak shape of 0.5). The median number of reported features in these studies is around 50,000, of which about a quarter to a third are considered high-quality features (SNR > 5, peak shape > 0.9). Because all organic molecular species contain both ^12^C and ^13^C (carbon 13, often under detection limit for low-abundance compounds) natural isotopologues, the presence of ^13^C/^12^C pair indicates high-confidence detection of a compound. The numbers of ^13^C/^12^C pairs are typically between 2,000 to 3,000 in these metabolomic datasets (Figure 1a). Signal intensity in a typical study follows a power law distribution (Figure 1b).

Typically, ESI is used in MS to avoid breaking up the molecules. In contrast, MS/MS techniques fragment molecules intentionally by a collision energy, and the fragments serve as a fingerprint of the molecule. If ISFs of a molecule are present in LC-MS data, they must a) have the same retention time as the intact molecules, and b) have m/z values that are similar to those in the corresponding MS/MS spectra. In Giera et al (2024), MS/MS data at zero collision energy were used to mimic MS data. We take a similar strategy here to compare an MS/MS database (MoNA2024) to the LC-MS metabolomics data, requiring both the precursor and at least one MS/MS fragment to be observed in MS data (Figure 1c). We parsed out 13,672 compounds from MoNA MS/MS spectra. They typically match to 5~10,000 features by precursor m/z values (Figure 1d). Of these matched precursor ions, about 7% have at least one matched MS/MS fragment in the elution window for positive ESI data, about 5% for negative ESI (Figure 1e). The elution window here is defined as two standard deviation of RT differences of all ^13^C/^12^C pairs. These results indicate that ISFs impact less than 10% of LC-MS features. The results are expected to include false positives (thus overestimation), as a) MS/MS fragments generated by higher collision energies are not expected to be in the LC-MS data; and b) matched features are not necessarily generated by ISF.

A limitation of the above approach is that MoNA database does not fully cover the LC-MS metabolomics data, though the number of matched MS/MS fragments relative to the matched precursor ions should indicate the prevalence of ISFs. A more systematic analysis can be performed using the khipu algorithm, which constructs ion patterns de novo into empirical compounds, in a process called pre-annotation (Li2023a, Mitchell2024). In short, a khipu aligns coeluting ions on a grid (a tree in computer term) of isotopologues and modifications, where modifications include adducts and fragments, as exemplified in Figure 2a. This approach depends on the list of mass differences underlying the ion patterns. We systematically calculate all common mass differences in these LC-MS datasets (Table S2–5). Not surprisingly, the m/z difference between ^13^C/^12^C isotopologues is by far the most frequent observation in both ESI+ and ESI- data. The top frequent mass differences, excluding known isotopic and adduct patterns, can be considered as candidates for ISFs (also referred as neutral losses, Figure 2b,c). Examining the RT shift of these candidate ISFs shows that the majority have identical RT as their molecular ions, confirming coelution in chromatography (Figure 2d).

To assess the impact of ISFs on full metabolomic profiles, we compare the pre-annotations without and with consideration of ISFs. Without ISFs, the khipu algorithm is applied to the LC-MS datasets using a conservative set of isotopologues and adducts (Methods), to produce a set of khipus and singletons per dataset. A singleton is a feature that has no known relationship to other features. The numbers of khipus and singletons can be impacted by ISFs, but ISFs do not impact relationships from isotopologues and adducts. Next, to consider ISFs, the candidate ISFs (from Figure 2b,c) are searched between khipus, and between singletons and khipus. The comparison between two steps reveals that in the ESI+ data, about 500 khipus and 700 singletons can be explained by the candidate ISFs (Figure 2e), which correspond to about 8% of khipus and 1% of all features, respectively (Figure 2f). In the ESI- data, the candidate ISFs explain about 5% of khipus and 1% of extra features (Figure 2e,f). While these results do not rule out additional ISFs among singleton features, singletons are of lower intensity and expected to contain even less percentage of fragments. The RT shift distributions in Figure 2d bear two shoulder peaks, which suggest that a subset of matched ISFs is not truly coeluting and thus not fragments but different compounds. This implies that the results in Figure 2e,f are an overestimation.

The khipu approach allows a full calculation of pre-annotation of all datasets now. A comprehensive set of isotopologues, adducts and ISFs are used in a new round of analysis (Table S6). The results show that about 16,000 features are explained by khipus in ESI+ or ESI- data, which correspond to 5~6,000 unique empirical compounds (Figure 3a). These numbers are in line with high-quality features in Figure 1a, suggesting that we are close to a full explanation of them. To test the dependency of pre-annotation on ion abundance, we split the features in each dataset by intensity into quartiles and visualize the pre-annotation for each quartile. The results show that about 60% of features in the top quartiles are explained by khipus in ESI+ or ESI- data, while the pre-annotated percentage is smaller in lower quartiles (Figure 3b). Plotting the result using full quantile range returns a similar conclusion, where pre-annotation reaches 80% at the top end of quantiles (Figure S1a). Within the top quartile, the singletons have significantly lower intensity than M0 features (Figure S1b), corroborating this observation that pre-annotation is bound by abundance. To test the impact of peak alignment on the results, we selected one random sample (thus avoiding peak alignment) from each dataset to repeat the full data processing and pre-annotation, which returned similar results (not shown).

In summary, we have employed two approaches to search ISFs in a sizable collection of LC-MS datasets. Both resulted in less than 10% of features being explained by ISFs. Additional analysis using 16 studies on time-of-flight platforms returned similar results (Tables S1, S7). If we assume ESI always fragments a small percentage of all analytes, our results are consistent with that by Giera et al (2024): in the analysis of authentic chemical standards, most ISFs are detected by mass spectrometer because there is little background and in the analysis of biological samples, the ISFs are outcompeted by more abundant molecular species in the complex matrix.

Overall, these results represent a broad survey of the current metabolomics data landscape: the khipu-based pre-annotation already explains the majority of abundant features. The less abundant features should not be considered as mystery - their analysis is just limited by concentration and ionization efficiency. Therefore, the “dark matter” of metabolomics is largely explainable. This means that the overwhelming majority of LC-MS metabolomic features from human blood samples are real compounds (not necessarily of biological origin). The differences between studies are often affected by experimental methods and conditions, which should be a focus of future computational data processing and annotation.

## Methods

### Data Retrieval and Processing

A total of 61 public metabolomics studies were downloaded from Metabolomics Workbench (metabolomicsworkbench.org) and MetaboLights (www.ebi.ac.uk/metabolights/). All studies were based on human serum or plasma samples, totaling 3482 samples. Of the 61 total studies, 45 were collected using orbitrap-type mass analyzers and 16 using time-of-flight mass analyzers. The breakdown by chromatography and ionization type are noted in Table S1 but all permutations of HILIC, RP, ESI+ and ESI- were represented in all analyzer subsets. Large studies were down selected to between 100~120 samples.

The raw files were converted to centroided mzML files using ThermoRawFileParser v1.3.1 for Orbitrap data or msConvert v3 for ToF data. Data preprocessing was performed using Asari (v 1.13.1), yielding feature tables with m/z, retention time, peak shape, signal-to-noise ratio, chromatographic selectivity, detection count and intensity values per study. For all analyses, the full feature table was used which enforces the default feature quality filters (signal-to-noise ratio, or SNR, of 2, and a peak shape > 0.5 defined as goodness of fit to a gaussian model). Orbitrap studies were processed using the autoheight option enabled, while ToF data was processed using a mass accuracy of 25 ppm, a min_peak_height of 1000, a cal_min_peak_height as 3e4, and a min_intensity_threshold of 500. Mass calibration was performed according to the default parameters in Asari.

The MoNA MS/MS library were downloaded from https://mona.fiehnlab.ucdavis.edu/ (Feb 8, 2024) and the resulting spectra were deduplicated as follows using utilities from MatchMS (Huber2024). First all spectra for the same precursor inchikey were identified to yield a spectral cluster. Within each spectral cluster, in the second step, the pairwise cosine similarity is calculated for all pairs. The sum of the cosine similarity score weighted by the number of matched peaks for all such comparisons per spectrum is then calculated. Lastly, the spectrum with the largest sum score for that inchikey is then selected as the exemplar for that inchikey and all others are discarded. All collision energy spectra were considered during deduplication with the assumption that the most common fragments should be frequently shared across collision energies.

### Search of m/z patterns

The isotopic pairs, ^13^C/^12^C features, are defined by annotation of matched retention time, mass distance of 1.003355, and intensity of ^13^C feature under 50% of that of ^12^C feature. The RT differences of all isotopic pairs in each dataset were calculated, and their standard deviation was used to define the RT coelution window per study (one standard deviation on either side of the molecular ion).

To compare MS/MS data to LC-MS data, our deduplicated MoNA MS/MS library is used (13973 precursors in positive and 9184 in negative). A 5-ppm mass tolerance was used for both precursor and MS/MS peak matching with features in retention time window. Only MS/MS peaks whose normalized intensity was higher than 0.1 was considered. To compare common mass difference to LC-MS data, in-source fragment candidates are manually selected from common mass differences, and the minimum of 5 ppm of m/z value or 0.0005 as absolute value was used for mass delta matching. Results here are searched on the top 5 MS/MS fragments, while using top 10 fragments led only to minor increase of matches (not shown).

### Calculation of common mass differences

For the molecular ion in each khipu, the m/z differences to all other features in the elution window were calculated and counted in a histogram (bin size 0.0001 for Orbitrap data, 0.0005 for TOF data). The histogram was smoothed, and peak values were selected by a threshold (100 for Orbitrap data, 20 for TOF data). The top 20 mass differences (deltas) were selected per ionization mode to be used as candidate in-source fragments.

### Pre-annotation using Khipu

**Figure 2:**
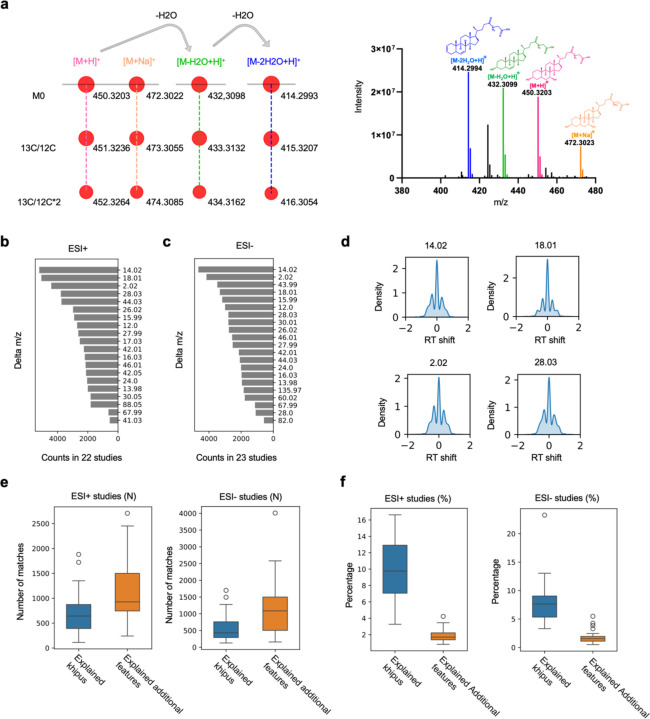
Most frequent neutral losses account for a small subset of LC-MS features. **a.** Example of khipu pre-annotation of isotopologues, adducts and fragments in a tree structure, with m/z values labeled for each ion. The mass spectrum is shown on the right. Data from sample 214 in study ST002112_RPpos_B3_ppm5_356194, RT around 619.49 second. This compound matches to glycochenodeoxycholic acid. **b**, **c**. Most frequence delta m/z values in positive and negative ionization datasets, respectively. These exclude obvious isotopologues and adducts, thus as candidates for fragments (neutral losses). **d**. Example distribution of retention time shift (in seconds) associated with each delta m/z in **b**. **e**, **f**. The numbers and percentages of khipus and additional features explained by candidate fragments, in positive and negative ionization datasets, respectively.

**Figure 3:**
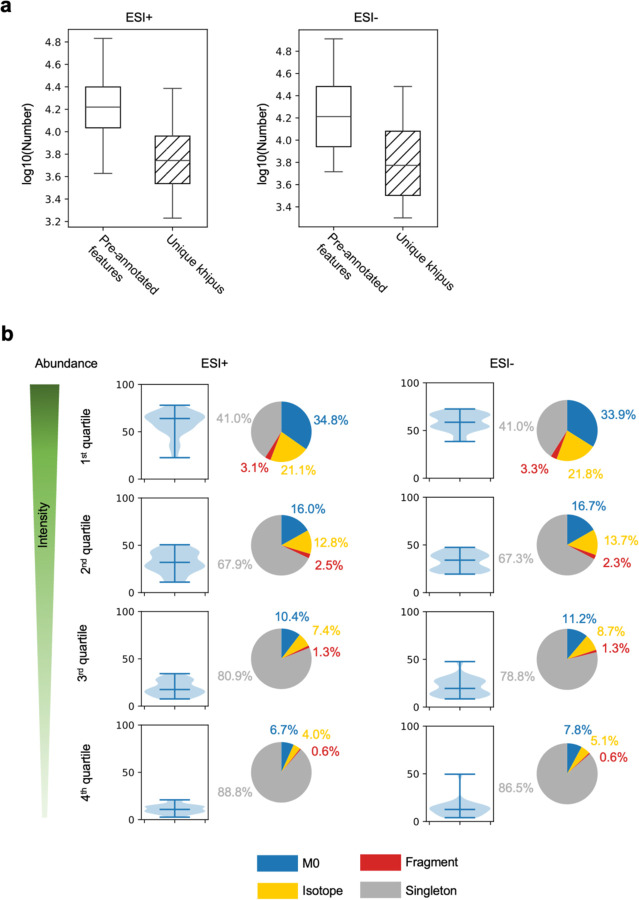
Khipu based pre-annotation explains most of the abundant features. **a.** Numbers of pre-annotated features cross studies, median value 16,574 and 16,296 for positive and negative ionization datasets, respectively. They correspond to 5,539 and 5,937 unique khipus, respectively. **b.** Percentage distribution of pre-annotated features in each intensity quartile (exemplified in Figure 1b) cross studies, shown as violin plots, for positive and negative ionization datasets respectively. The pie charts show median percentage of contributions from M0, other isotopologues, in-source fragments and singletons. Adducts are included in khipus, and singletons are the unexplained features.

Khipu performs pre-annotation by assigning co-eluting features to adduct and isotopologue relations using a generic tree structure based on *a priori* mass delta patterns (Li2023a). The minimum of 5 ppm of m/z value or 0.0005 as absolute value was used for mass delta matching. The conservative parameters used in Figure 2 are as follows: in positive ionization mode, isotope patterns 13C/12C at m/z 1.0034, 13C/12C × 2 at m/z 2.0067, and 37Cl/35Cl at m/z 1.9970; adducts Na/H at m/z 21.9819, ACN at m/z 41.0265, NaCOOH at m/z 67.9874, K/H at m/z 37.9559, and CH3OH at m/z 32.0262. In negative ionization mode, isotope patterns 13C/12C at m/z 1.0034, 13C/12C × 2 at m/z 2.0067, 37Cl/35Cl at m/z 1.9970, and 32S/34S at m/z 1.9958; adducts Na/H at m/z 21.9819, NaCOOH at m/z 67.9874, and NaCH2COOH at m/z 82.0030. The parameters for comprehensive khipu analysis in Figure 3 are given in Table S6.

## Supplementary Material

Supplement 1 Table S1. List of datasets used in this work, include 45 Orbitrap studies and 16 TOF studies.
Table S2. Most frequent mass differences in Orbitrap ESI+ data.
Table S3. Most frequent mass differences in Orbitrap ESI- data.
Table S4. Most frequent mass differences in TOF ESI+ data.
Table S5. Most frequent mass differences in TOF ESI- data.
Table S6. Isotopologues, adducts and ISFs used for extended khipu search in Figure 3.
Table S7. Results of features explained in TOF LC-MS metabolomics data.

Supplement 2 Figure S1: Dependency of pre-annotation on feature abundance.

## Figures and Tables

**Figure 1.**
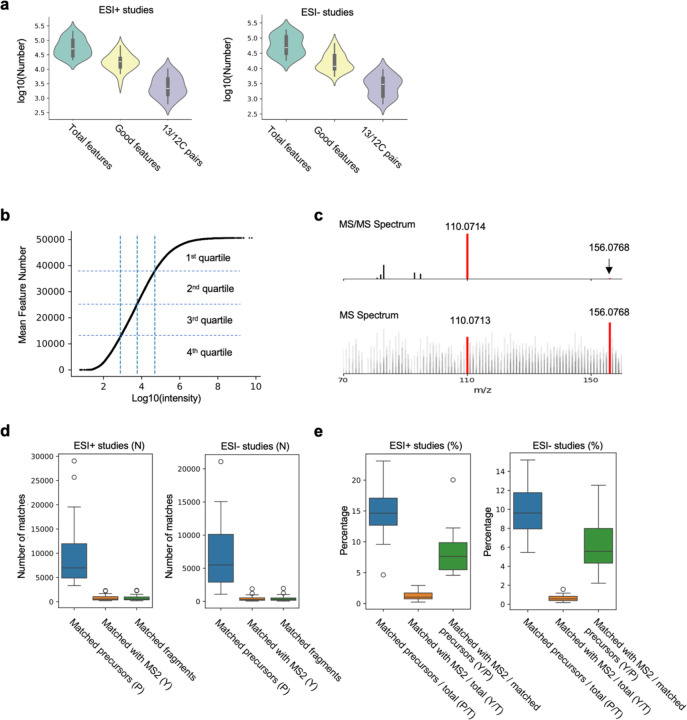
Search of in-source fragments in 45 LC-MS studies from Orbitrap platforms using MS/MS spectra. **a.** Across the 22 studies using positive ionization, the median number of features is 50,771, the median number was 18,117, and the number of matched ^13^C/^12^C pairs was 2,176. Similar numbers were observed across the 23 negative ionization studies with 48,212 features, 11,783 good features, and 3,008 ^13^C/^12^C pairs. Good features are defined as having SNR > 5 and peak shape > 0.9 when fitting to a gaussian curve. **b.** Example distribution of feature intensity (dataset ST002200_RPpos_17min_B3_ppm5_3422144, total 50,647 features). Quartiles are marked by dashed lines. **c.** Example of matching MS/MS and MS spectra for searching ISFs. Top: MS/MS from MoNA. Bottom: sample CHCL3_20min_B from dataset MTBLS1465_HILICpos_ppm5_3505731, MS scan number 1408. **d.** Absolute numbers of features matched to MoNA MS/MS spectra across the 45 LC-MS studies, using positive and negative ionization respectively. In each boxplot, 1^st^ column is the number of features matching a precursor m/z value (P), the 2^nd^ column the subset of P with match to at least one MS/MS fragment (Y), and the 3^rd^ column number of all matched MS/MS fragments per study. **e.** Conversion of **d** to percentages: P over total features (T), Y over T and Y over P.

## Data Availability

All asari processed data are available at: https://zenodo.org/records/14541717. The lists of mass patterns for isotopologues, adducts and ISFs are included in the mass2chem software package, freely available at https://github.com/shuzhao-li-lab/mass2chem. All data analysis in this work is provided as Jupyter notebooks at https://github.com/shuzhao-li-lab/in_source_fragments_serum.
