# Systematic pre-annotation explains the “dark matter” in LC-MS metabolomics

**DOI:** 10.1101/2025.02.04.636472

**Published:** 2025-03-25

**Authors:** Yuanye Chi, Joshua M. Mitchell, Shujian Zheng, Shuzhao Li

**Affiliations:** 1 The Jackson Laboratory for Genomic Medicine, 10 Discovery Drive, Farmington, CT 06032, USA; 2 University of Connecticut School of Medicine, Farmington, CT 06032, USA

## Abstract

The majority of features in global metabolomics from high-resolution mass spectrometry are typically not identified, referred as the “dark matter”. Are these features real compounds or junk? Understanding this problem is critical to the annotation and interpretation of metabolomics data and future development of the field. Recent debates also brought attention to in-source fragments, which appear to be prevalent in spectral databases. We report here a systematic analysis of 61 representative public datasets from LC-MS metabolomics, the most common data type in biomedical studies. The results indicate that in-source fragments contribute to less than 10% of features in LC-MS metabolomics. Khipu-based pre-annotation shows that majority of abundant features have identifiable ion patterns. This suggests that the “dark matter” in LC-MS metabolomics is explainable in an abundance dependent manner; most features are from real compounds; the number of compounds is much smaller than that of features; most compounds are yet to be identified.

The “dark matter” of metabolomics refers to the large number of unidentified features in metabolomic studies, mostly from mass spectrometry (MS) based analysis (deSilva2015; Monge2019). The topic is pertinent to the analytical coverage of small molecules in biomedical research (Kind2009; Uppal2016), approaches to metabolite annotation (Domingo2018; Chaleckis2019; Metz2025), mapping reaction pathways (Zamboni2015; Artyukhin2018) and the promise of applying metabolomics and exposomics to precision medicine (Wishart2016; Vermeulen2020; David2021). Multiple factors contribute to this “dark matter”. Modern MS brings tremendous new power into chemical analysis, enabling the detection of compounds that were previously elusive due to limited separation, abundance or stability (Xian2012; Uppal2016; Lai2024). Biology has knowledge gaps and new biochemical mechanisms continue to be discovered. E.g. hundreds of unknown metabolites can be affected by one enzyme (Artyukhin2018; Heremans2022). Such results suggest that characteristic metabolite modifications may be as widespread as protein posttranslational modifications. Our cohabitation with the microbiome results in sharing many metabolites from under-characterized microbial species (Peisl2018). Challenges exist in data acquisition, processing and interpretation.

Contamination is common in tandem mass spectra (MS/MS) from biological samples (Stancliffe2021). Artifacts can be generated by data processing software when sensitivity is not matched to detection confidence (Li2023b). Because isotopologues, adducts and fragments are commonly measured in the MS data, it is an apt question if the measured metabolome is inflated by degenerate features (Mahieu2017; Wang2019; Li2023a).

In-source fragments (ISFs), generated when molecules are broken down by ionization energy, became a focus of recent debates. Giera et al (2024) reported that ISFs accounted for over 70% of MS/MS features in METLIN, one of the leading spectral databases, suggesting that ISFs could be a significant portion of the “dark matter”. El Abiead et al (2025) calculated the presence of ISFs using a different compound library, reporting less percentage but still two fragments on average per protonated compound. However, both studies focused on data from chemical standards, which are primarily used for metabolite annotation. Currently, the majority of metabolomics data in biomedical research are produced using LC-MS (liquid chromatography coupled mass spectrometry). Biological samples are complex: a LC-MS spectrum often contains more than 1000 mass peaks while a MS/MS spectrum of chemical standards has usually less than 100. It is common in LC-MS that over 1000 spectra are collected on one sample during the chromatographic separation. Therefore, feature detection in LC-MS data requires consecutive elution patterns, a quality filter removing many inconsistent data points. The LC-MS data from biological samples are very different from that of chemical standards. The real number of metabolites and impact of ISFs should be assessed directly on biological data.

Here, we report a systematic analysis of LC-MS metabolomics from 61 representative public datasets. The relationships of isotopologues, adducts and fragments are determined by mass differences between coeluting features, in a process termed “pre-annotation” (Li2023a). Our results indicate that the contribution of ISFs is small in LC-MS metabolomics, but majority of abundant features have identifiable ion patterns. This suggests that the “dark matter” in metabolomics is explainable in an abundance dependent manner.

## Results

### LC-MS metabolomics data have a long tail of low intensity features

**Figure 1.**
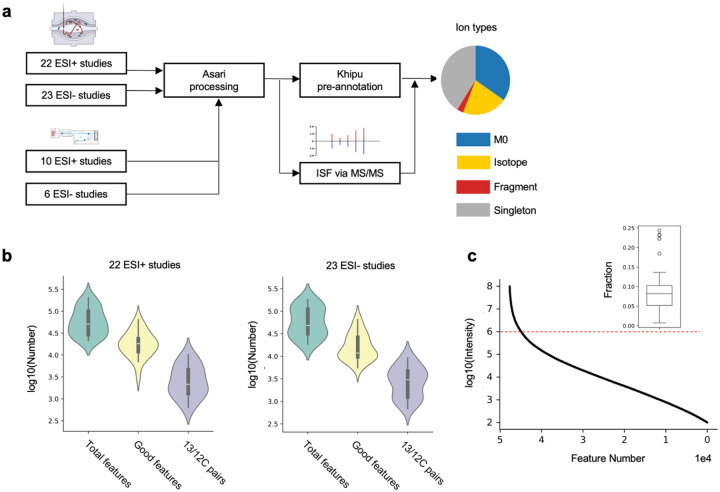
Processing and analysis of 61 LC-MS studies **a.** Schematic of pre-annotation analysis, from orbital or time-of-flight instruments. ESI: electron spray ionization; ISF: in-source fragment. M0: the isotopic form of ^12^C only. The results from 16 TOF datasets are shown in supplements. **b.** Across the 22 Orbitrap studies using positive ionization, the median number of features is 50,771, the median number of good features 18,117, and the number of matched ^13^C/^12^C pairs 2,176. Similar numbers are observed across the 23 negative ionization Orbitrap studies with 48,212 features, 11,783 good features, and 3,008 ^13^C/^12^C pairs. Good features are defined as having SNR > 5 and peak shape > 0.9 when fitting to a gaussian curve. **c.** Long-tail distribution of feature intensity, where intensity is plotted at log10 scale. Example from dataset ST002200_RPpos_17min_B3_ppm5_3422144, total 50,647 features. Insert boxplot is the fraction of features above 1E6 in intensity, cross 22 Orbitrap ESI+ datasets.

High-resolution mass spectrometers, with electron spray ionization (ESI), are now routinely employed in metabolomics. From public repositories, we retrieved 61 LC-MS metabolomics datasets. These include 45 studies from orbital mass analyzers (Orbitrap) and 16 from time-of-flight (TOF) analyzers, using in positive or negative ionization (Figure 1a, Table S1). For consistence of data analysis, only studies of human plasma or serum samples are included, and large datasets are down selected to about 100 samples, because low-frequency features accumulate by increasing sample number. We use the asari software (Li2023b) for processing raw data to features of unique m/z (mass-to-charge ratio) and retention time (RT). Asari is designed with explicit data models that report all features above a quality threshold (signal-to-noise ratio, or SNR, of 2, peak shape of 0.5). The median number of reported features in the Orbitrap studies is around 50,000, of which about a quarter to a third are considered high-quality features (SNR > 5, peak shape > 0.9). Because all organic molecular species contain both ^12^C and ^13^C (carbon 13, often under detection limit for low-abundance compounds) natural isotopologues, the presence of ^13^C/^12^C pair indicates high-confidence detection of a compound. The numbers of ^13^C/^12^C pairs are typically between 2,000 to 3,000 in these metabolomic datasets (Figure 1b). Signal intensity in a typical study follows a long-tail distribution, where about 9% features have higher intensity than 1E6 (Figure 1c).

### Organizing peaks into putative compounds

**Figure 2:**
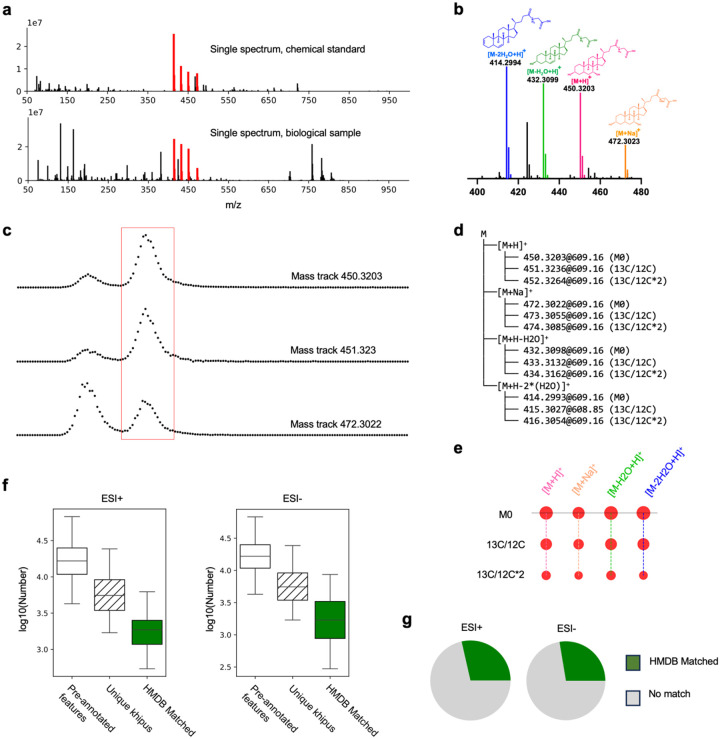
LC-MS metabolomics features explained by khipu pre-annotation. **a.** Full scan mass spectra of glycochenodeoxycholic acid, analyzed from chemical standard (top, containing impurities) and observed in a biological sample (bottom. Sample 214 in study ST002112_RPpos_B3_ppm5_356194, RT 619.49 second). This is chosen to illustrate a complex ion pattern, while most metabolites have fewer ions. MS/MS data can be generated on a mass peak here, but are not included in this figure. **b.** Manual interpretation of the mass peaks (red in **a**). Smaller peaks of the same color are M2 and M3 isotopologues, which are difficult to see in the full mass range in **a**. **c.** Example mass tracks (extracted ion chromatogram) from same biological sample. Elution profiles are composed from consecutive spectra (scan numbers 600–650), each dot from one spectrum. Red box marks the LC-MS features related to glycochenodeoxycholic acid. Reported retention time may be adjusted by alignment cross samples. **d.** Example of khipu pre-annotation of isotopologues, adducts and fragments in a tree structure, with m/z values labeled for each ion. **e.** Khipugram visualization of the data in **d**. Khipu is both the name of software and of a group of ions belonging a putative compound. **f.** Numbers of pre-annotated features cross studies, median value 16,574 and 16,296 for positive and negative ionization datasets, respectively. They correspond to 5,539 and 5,937 unique khipus, of which 1866 and 1692 matched to HMDB version 5 by neutral mass, respectively. **g.** Average matches of khipus to HMDB are 31.8% and 28.6%, for positive and negative ionization datasets, respectively.

The number of features is not a direct account of number of compounds, due to the existence of isotopologues, adducts and fragments. The complexity is illustrated for a compound analyzed from its chemical standard and from a biological sample (MS profiles, Figure 2a). This compound shows mass peaks for two adducts, two neutral losses of H_2_O and three isotopologues (Figure 2b). It is important to note that features of interest in a spectrum are mass peaks, while in the current practice of LC-MS, features are elution peaks that are observed in consecutive spectra (Figure 2c). There is a major reduction of data complexity by feature detection in LC-MS; the complexity of redundant features is then reduced by the process of pre-annotation.

We have previously described a software algorithm, khipu, to perform de novo, global pre-annotation (Li2023a, Mitchell2024b). In short, a khipu aligns coeluting ions on a grid (a tree in computer term) of isotopologues and modifications, where modifications include adducts and fragments (Figure 2d,e). Conjugates of compounds (multimers) are less common and not included here. If a feature finds no relationship to other ions, it is called a singleton. The khipu construction leads to an inferred neutral mass, and the data structure enables downstream computing. Not all khipus have multiple adducts, but the ^13^C ion is expected to be present for all organic compounds of reasonable abundance. Therefore, a khipu indicates a bona fide compound (present in the sample, not necessarily of biological origin). The number of khipus is the estimate of number of high-confidence compounds in the data.

Applying this pre-annotation to all datasets reveals that each dataset typically contains 5~6,000 khipus, which explain about 16,000 features in ESI+ or ESI− data (Figure 2f). The average khipu contains three features (the compound shown in Figure 2a–e is atypical). These numbers are in line with high-quality features in Figure 1b, suggesting that we are close to a full explanation of them. We next compare the neutral mass values of khipus to HMDB (version 5, Wishart2022), the leading metabolite database. On average, about 30% of these khipus are matched to HMDB, indicating the majority of compounds in these studies are unknown (Figure 2g).

### Common mass differences in LC-MS and application to ISF estimation

**Figure 3:**
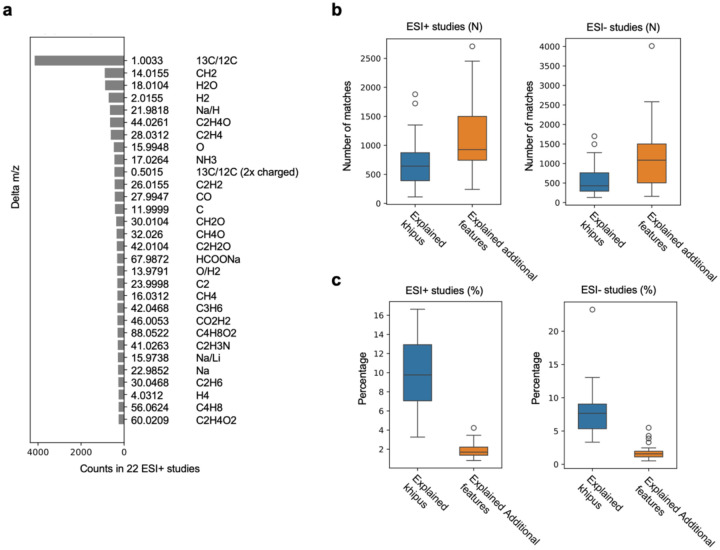
Most frequent neutral losses account for a small subset of LC-MS features. **a**. Most frequence delta m/z values in positive ionization Orbitrap datasets. **b**, **c**. The numbers and percentages of khipus and additional features explained by candidate fragments, in positive and negative ionization datasets, respectively.

The pre-annotation approach depends on the list of mass differences underlying the ion patterns. The khipu software includes default lists of isotopes and adducts based on prior data. To be comprehensive, we also systematically calculated all common mass differences between coeluting features in these current LC-MS datasets (Table S2–5). The coelution window here is defined as two standard deviation of RT differences of all ^13^C/^12^C pairs. Not surprisingly, the m/z difference between ^13^C/^12^C isotopologues is by far the most frequent observation in both ESI+ and ESI− data (Figures 3a, S1a). The top frequent mass differences, excluding known isotopic and adduct patterns, can be considered as candidates for ISFs (also referred as neutral losses).

To assess the impact of ISFs on full metabolomic profiles, we compare the pre-annotations without and with consideration of ISFs. Without ISFs, the khipu algorithm is applied to the LC-MS datasets using a conservative set of isotopologues and adducts (Methods), to produce a set of khipus and singletons per dataset. The numbers of khipus and singletons can be impacted by ISFs, but ISFs do not impact relationships between isotopologues and adducts. Next, to consider ISFs, the candidate ISFs (Table S6, Figures 3a, S1a) are searched between khipus, and between singletons and khipus. The comparison between two steps reveals that in the ESI+ data, about 500 khipus and 700 singletons can be explained by the candidate ISFs (Figure 3b), which correspond to about 8% of khipus and 1% of all features, respectively (Figure 3c). In the ESI− data, the candidate ISFs explain about 5% of khipus and 1% of extra features (Figure 3b,c). While these results do not rule out additional ISFs among singleton features, singletons are of lower intensity and expected to contain even less percentage of fragments.

The above approach is an approximation. Examining the RT shift of these candidate ISFs shows that the majority have identical RT as their molecular ions, confirming coelution in chromatography (Figure S1b). The RT shift distributions in Figure s1b bear two shoulder peaks, which suggest that a subset of matched ISFs is not truly coeluting, thus not fragments but different compounds. This can lead to overestimation in Figure 3b,c. The candidate ISFs are not exhaustive, not considering chemical structures in their preference of fragmentation, which could lead to minor underestimation. We next investigate ISFs using a different approach via MS/MS spectra.

### MS/MS based search also shows less than 10% ISFs in LC-MS data

**Figure 4:**
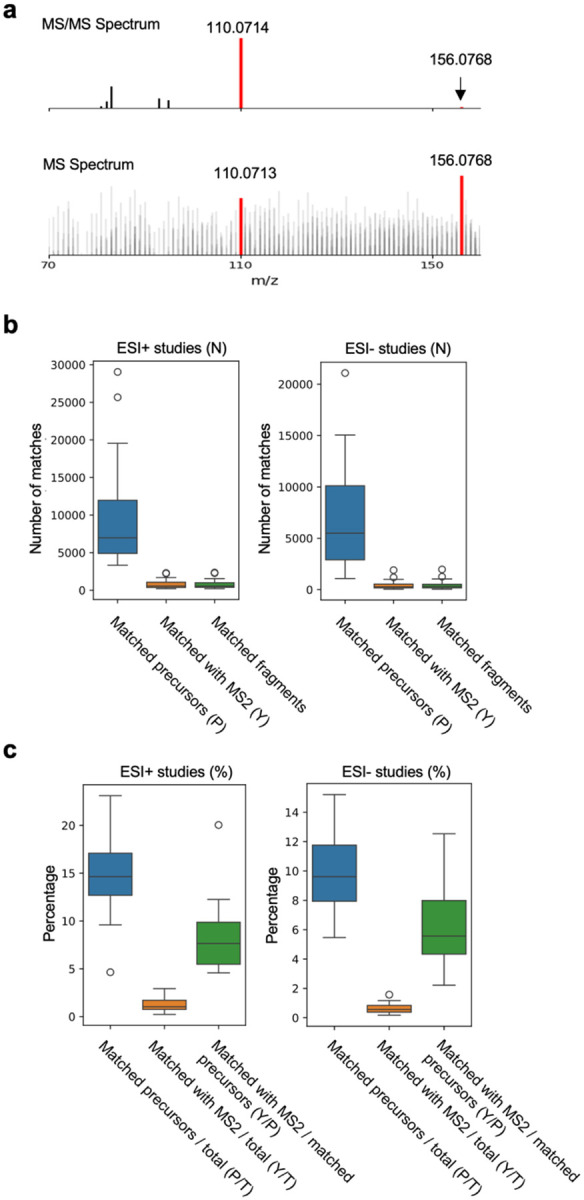
MS/MS based search of ISFs. **a.** Example of matching MS/MS and MS spectra for searching ISFs. Top: MS/MS from MoNA. Bottom: sample CHCL3_20min_B from dataset MTBLS1465_HILICpos__ppm5_3505731, MS scan number 1408. **b.** Absolute numbers of features matched to MoNA MS/MS spectra across the 45 LC-MS studies, using positive and negative ionization respectively. In each boxplot, 1^st^ column is the number of features matching a precursor m/z value (P), the 2^nd^ column the subset of P with match to at least one MS/MS fragment (Y), and the 3^rd^ column number of all matched MS/MS fragments per study. **c.** Conversion of **b** to percentages: P over total features (T), Y over T and Y over P.

Typically, ESI is used in MS to avoid breaking up the molecules. In contrast, MS/MS techniques fragment molecules intentionally by a collision energy, and the fragments serve as a fingerprint of the molecule. If ISFs of a molecule are present in LC-MS data, they must a) have the same retention time as the intact molecules, and b) have m/z values that are similar to those in the corresponding MS/MS spectra. In Giera et al (2024), MS/MS data at zero collision energy were used to mimic MS data. We take a similar strategy here to compare an MS/MS database (MoNA2024) to the LC-MS metabolomics data, requiring both the precursor and at least one MS/MS fragment to be observed in MS data (Figure 4a). Even though MoNA database does not fully cover the LC-MS metabolomics data, the number of matched MS/MS fragments relative to the matched precursor ions should indicate the prevalence of ISFs. We parsed out 13,672 compounds from MoNA MS/MS spectra. They typically match to 5~10,000 features by precursor m/z values (Figure 4b). Of these matched precursor ions, about 7% have at least one matched MS/MS fragment in the elution window for positive ESI data, about 5% for negative ESI (Figure 4c). These results confirm the above khipu analysis that ISFs impact less than 10% of LC-MS features. The results are expected to include false positives (thus overestimation), as a) MS/MS fragments generated by higher collision energies are not expected to be in the LC-MS data; and b) matched features are not necessarily generated by ISF.

### An abundance dependent explanation of the “dark matter “

**Figure 5:**
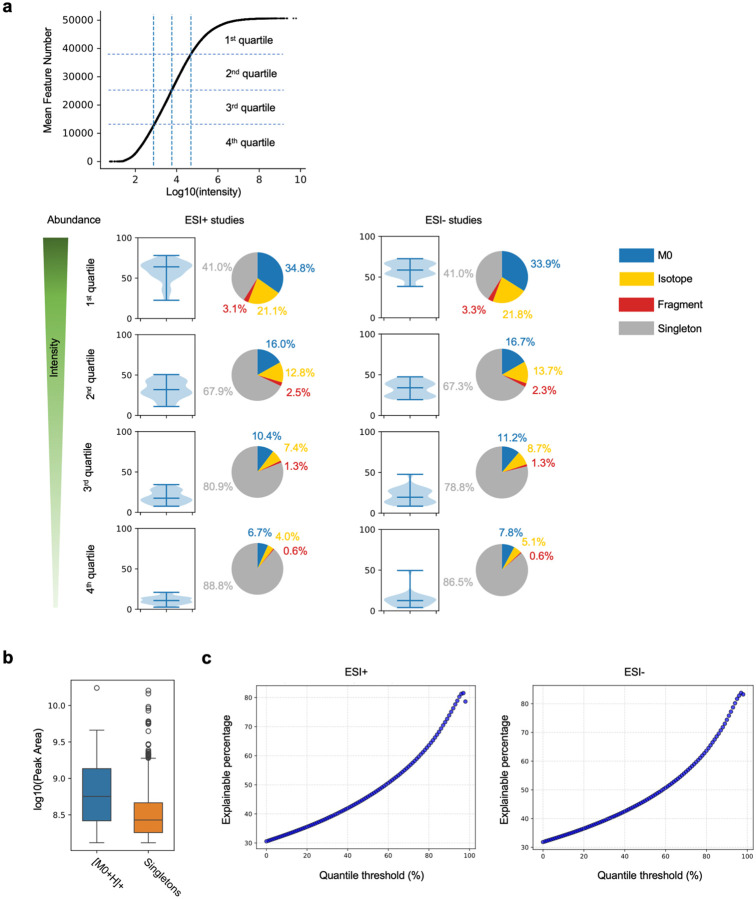
Khipu based pre-annotation explains most of the abundant features. **a.** Top: Distribution of feature intensity in Figure 1c divided into quartiles, which are marked by dashed lines. Bottom: Percentage distribution of pre-annotated features in each intensity quartile cross studies, shown as violin plots, for positive and negative ionization datasets respectively. The pie charts show median percentage of contributions from M0, other isotopologues, in-source fragments and singletons. Adducts are included in khipus, and singletons are the unexplained features. **b.** Abundance difference between [M0+H]+ features and singletons within the top quartile. Example from study ST001237_HILICpos_B2_ppm5_3524314, p-value < 1E-25 by Student t-test. **c.** Percentage of pre-annotated features versus feature abundance quantile (1%−99% stepwise by 1%) from 45 Orbitrap studies, for positive and negative ionization datasets respectively.

While the numbers of features explained by khipus are given in Figure 2f, we are reminded that high-abundance features are a small fraction of all data (Figure 1c). To test the dependency of pre-annotation on feature abundance, we split the features in each dataset by intensity into quartiles and visualize the pre-annotation for each quartile. The results show that about 60% of features in the top quartiles are explained by khipus in ESI+ or ESI− data, while the pre-annotated percentage is smaller in lower quartiles (Figure 5a). Within the top quartile, the singletons, i.e., features not explained by khipus, have significantly lower intensity than M0 features (Figure 5b), corroborating the observation that pre-annotation is bound by abundance. Plotting the result using full quantile range returns a similar conclusion, where pre-annotation reaches 80% at the top end of quantiles (Figure 5c). The analysis using 16 studies on time-of-flight platforms returned similar results (Tables S1, S7). To test the impact of peak alignment on the results, we selected one random sample (thus avoiding peak alignment) from each dataset to repeat the full data processing and pre-annotation, which returned similar results (not shown).

## Discussion

In summary, we have carried out systematic pre-annotation on 61 LC-MS metabolomics datasets, representing a reasonable survey of the current data landscape. The results from two different approaches indicate that less than 10% of features in LC-MS are explained by ISFs, while the number may vary between studies. On the surface, this is quite different from the numbers reported by Giera et al (2024) and El Abiead et al (2025). However, results from all three studies have a consistent explanation. ESI can generate many fragments but of low intensity (also pointed out by El Abiead et al, 2025). In the analysis of authentic chemical standards, many ISFs are detected by mass spectrometer because there is little background, while the percentage varies by platforms. There are often over 1 million mass peaks per sample in LC-MS metabolomics, where data are processed into features based on elution peaks. Most ISFs are not observed in LC-MS feature detection because a) signals inconsistent cross scans are filtered out by elution peaks, and b) low intensity ISFs are outcompeted by more abundant molecular species in the complex matrix of biological samples.

Is the metabolomics “dark matter” real metabolites or junk? The majority of abundant features in LC-MS are already explained by the khipu-based pre-annotation. This indicates that the interpretation of less abundant features is just limited by concentration and ionization efficiency. Therefore, the “dark matter” of metabolomics is largely explainable and should not be considered as mystery. The corollary is that the overwhelming majority of LC-MS metabolomic features from human blood samples are of real compounds. The number of compounds is much smaller than the number of features. Most of these compounds are not matched in HMDB and considered unknown. We do not assume all the compounds are of biological origin. A compound should only be considered as a biological metabolite based on evidence of its activity.

Several precautions may protect us from confusions in the field. Metabolomics moves the traditional analytical chemistry to the -omics scale of complex data. Statistics in spectral databases is difficult to extrapolate directly into LC-MS metabolomics on biological samples. Explicit data models are important to define the purpose and context of results; and analyzing complex data calls for reusable software tools and code (Mitchell2024a). There are many data points and low-quality features in metabolomics data, easily leading to artifacts in computational processing (Li2023b). Different from calling nucleotide bases in genomics, metabolomics features are analog signals that require explicit quality metrics. There are clear benefits to separate pre-annotation from annotation. In genomics, a gene sequence can be deposited for many years before annotation is assigned. While the metabolomics community continues working on compound identification, pre-annotation already answers import questions. The inflation of compound numbers in database searches can be much reduced by using neutral mass from pre-annotation, not individual m/z features.

The size of metabolome depends on the depth and coverage of analysis. Detection frequency across samples also varies greatly on compounds, raising the question of distinguishing metabolome from exposome (Banbury2025). This study does not address how the coverage of different datasets is affected by experimental methods and conditions, which should be a focus of future computational data processing and annotation.

## Methods

### Data Retrieval and Processing

A total of 61 public, untargeted metabolomics studies were downloaded from Metabolomics Workbench (metabolomicsworkbench.org) and MetaboLights (www.ebi.ac.uk/metabolights/). All studies were based on human serum or plasma samples, totaling 3482 samples. Of the 61 total studies, 45 were collected using orbitrap-type mass analyzers and 16 using time-of-flight mass analyzers. The breakdown by chromatography and ionization type are noted in Table S1 but all permutations of HILIC, RP, ESI+ and ESI− were represented in all analyzer subsets. Large studies were down selected to between 100~120 samples.

The raw files were converted to centroided mzML files using ThermoRawFileParser v1.3.1 for Orbitrap data or msConvert v3 for ToF data. Data preprocessing was performed using Asari (v 1.13.1), yielding feature tables with m/z, retention time, peak shape, signal-to-noise ratio, chromatographic selectivity, detection count and intensity values per study. For all analyses, the full feature table was used which enforces the default feature quality filters (signal-to-noise ratio, or SNR, of 2, and a peak shape > 0.5 defined as goodness of fit to a gaussian model). Orbitrap studies were processed using the autoheight option enabled, while ToF data was processed using a mass accuracy of 25 ppm, a min_peak_height of 1000, a cal_min_peak_height as 3e4, and a min_intensity_threshold of 500. Mass calibration was performed according to the default parameters in Asari.

The MoNA MS/MS library were downloaded from https://mona.fiehnlab.ucdavis.edu/ (Feb 8, 2024) and the resulting spectra were deduplicated as follows using utilities from MatchMS (Huber2024). First all spectra for the same precursor inchikey were identified to yield a spectral cluster. Within each spectral cluster, in the second step, the pairwise cosine similarity is calculated for all pairs. The sum of the cosine similarity score weighted by the number of matched peaks for all such comparisons per spectrum is then calculated. Lastly, the spectrum with the largest sum score for that inchikey is then selected as the exemplar for that inchikey and all others are discarded. All collision energy spectra were considered during deduplication with the assumption that the most common fragments should be frequently shared across collision energies.

### LC-MS analysis of glycochenodeoxycholic acid

The chemical standard of glycochenodeoxycholic acid (Cayman Chemical, catalog number 16942) was prepared with acetonitrile at 1 μg/mL. The standard was run using a HILIC column on a Thermo Scientific Q Exactive HF-X Mass Spectrometer (Thermo Fisher Scientific, MA, USA) coupled to Thermo Scientific Transcen LX-2 Duo UHPLC system (Thermo Fisher Scientific, MA, USA), with a HES-II ionization source. The HILIC method utilized an Accucore^™^ 150 Amide column (10×2.1 mm guard; 100×2.1 mm analytical) with 10 mM ammonium acetate mobile phases (95:5 acetonitrile:water, v/v) containing 0.1% acetic acid, with a gradient from 0% to 98% B2 over 20 minutes at a flow rate of 0.55 mL/min and 45 °C column temperature. MS data were acquired in positive ionization modes (66.7–1000.0 m/z, resolution 60,000 at m/z 200) with optimized ion source parameters, including 3.5 kV spray voltage, 300 °C capillary temperature, and 425 °C heater temperature.

### Search of m/z patterns

The isotopic pairs, ^13^C/^12^C features, are defined by annotation of matched retention time, mass distance of 1.003355, and intensity of ^13^C feature under 50% of that of ^12^C feature. The RT differences of all isotopic pairs in each dataset were calculated, and their standard deviation was used to define the RT coelution window per study (one standard deviation on either side of the molecular ion).

To compare MS/MS data to LC-MS data, our deduplicated MoNA MS/MS library is used (13973 precursors in positive and 9184 in negative). A 5-ppm mass tolerance was used for both precursor and MS/MS peak matching with features in retention time window. Only MS/MS peaks whose normalized intensity was higher than 0.1 was considered. To compare common mass difference to LC-MS data, in-source fragment candidates are manually selected from common mass differences, and the minimum of 5 ppm of m/z value or 0.0005 as absolute value was used for mass delta matching. Results here are searched on the top 5 MS/MS fragments, while using top 10 fragments led only to minor increase of matches (not shown).

### Calculation of common mass differences

For the molecular ion in each khipu, the m/z differences to all other features in the elution window were calculated and counted in a histogram (bin size 0.0001 for Orbitrap data, 0.0005 for TOF data). The histogram was smoothed, and peak values were selected by a threshold (100 for Orbitrap data, 20 for TOF data). The top 20 mass differences (deltas) were selected per ionization mode to be used as candidate in-source fragments.

### Pre-annotation using Khipu

Khipu performs pre-annotation by assigning co-eluting features to adduct and isotopologue relations using a generic tree structure based on *a priori* mass delta patterns (Li2023a). The minimum of 5 ppm of m/z value or 0.0005 as absolute value was used for mass delta matching. The conservative parameters used in Figure 3 without ISFs are as follows: in positive ionization mode, isotope patterns 13C/12C at m/z 1.0034, 13C/12C × 2 at m/z 2.0067, and 37Cl/35Cl at m/z 1.9970; adducts Na/H at m/z 21.9819, ACN at m/z 41.0265, NaCOOH at m/z 67.9874, K/H at m/z 37.9559, and CH3OH at m/z 32.0262. In negative ionization mode, isotope patterns 13C/12C at m/z 1.0034, 13C/12C × 2 at m/z 2.0067, 37Cl/35Cl at m/z 1.9970, and 32S/34S at m/z 1.9958; adducts Na/H at m/z 21.9819, NaCOOH at m/z 67.9874, and NaCH2COOH at m/z 82.0030. The parameters for comprehensive khipu analysis in Figures 2 and 5 are given in Table S6.

## Supplementary Material

Supplement 1

1

## Data Availability

All asari processed data are available at: https://zenodo.org/records/14541717. The lists of mass patterns for isotopologues, adducts and ISFs are included in the mass2chem software package, freely available at https://github.com/shuzhao-li-lab/mass2chem. All data analysis in this work is provided as Jupyter notebooks at https://github.com/shuzhao-li-lab/in_source_fragments_serum.
