# Supplementary material for: Systematic pre-annotation explains the “dark matter” in LC-MS metabolomics
Source: bioRxiv. 2025 Mar 25:2025.02.04.636472. Originally published 2025 Feb 5. Preprint. [Version 2] doi: 10.1101/2025.02.04.636472 (PMC11838597; doi:10.1101/2025.02.04.636472)

bioRxiv preprint doi: https://doi.org/10.1101/2025.02.04.636472; this version posted March 25, 2025. The copyright holder for this preprint (which was not certified by peer review) is the author/funder, who has granted bioRxiv a license to display the preprint in perpetuity. It is made available under aCC-BY 4.0 International license.

Figure S1: Additional common mass differences and distribution of retention shift.

a. Most frequence delta m/z values in negative ionization Orbitrap datasets.

b. Example distribution of retention time shift (in seconds) associated with each delta m/z (labeled at top) in **Figure 3a**.

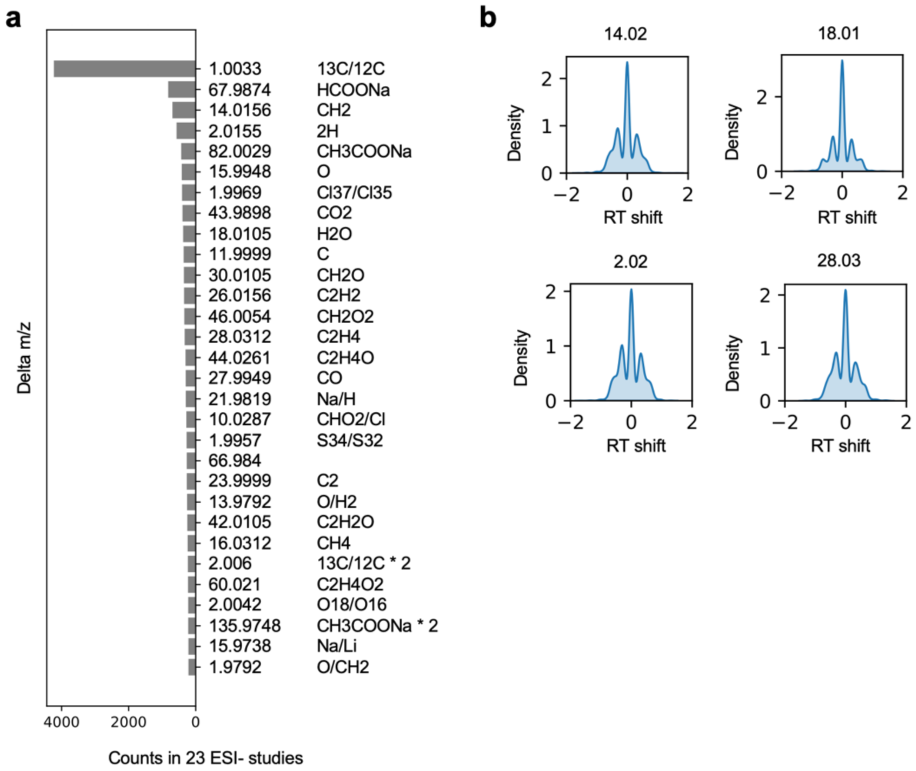

Supplement: 1 [file NIHPP2025.02.04.636472V2-supplement-1.pdf]
