# Supplementary material for: A systematic analysis of in-source fragments in LC-MS metabolomics
Source: bioRxiv. 2025 Feb 5:2025.02.04.636472. Preprint. [Version 1] doi: 10.1101/2025.02.04.636472 (PMC11838597; doi:10.1101/2025.02.04.636472)

bioRxiv preprint doi: https://doi.org/10.1101/2025.02.04.636472; this version posted February 5, 2025. The copyright holder for this preprint (which was not certified by peer review) is the author/funder, who has granted bioRxiv a license to display the preprint in perpetuity. It is made available under aCC-BY 4.0 International license.

Figure S1: Dependency of pre-annotation on feature abundance.

a. Percentage of pre-annotated features versus feature quantile by peak area (1%-99% with stepwise as 1%) from 45 Orbitrap studies, for positive and negative ionization datasets respectively.

b. Example of abundance difference between [M0+H]+ features and singletons from study ST001237_HILICpos_B2_ppm5_3524314, p-value < 1E-25 by Student t-test.

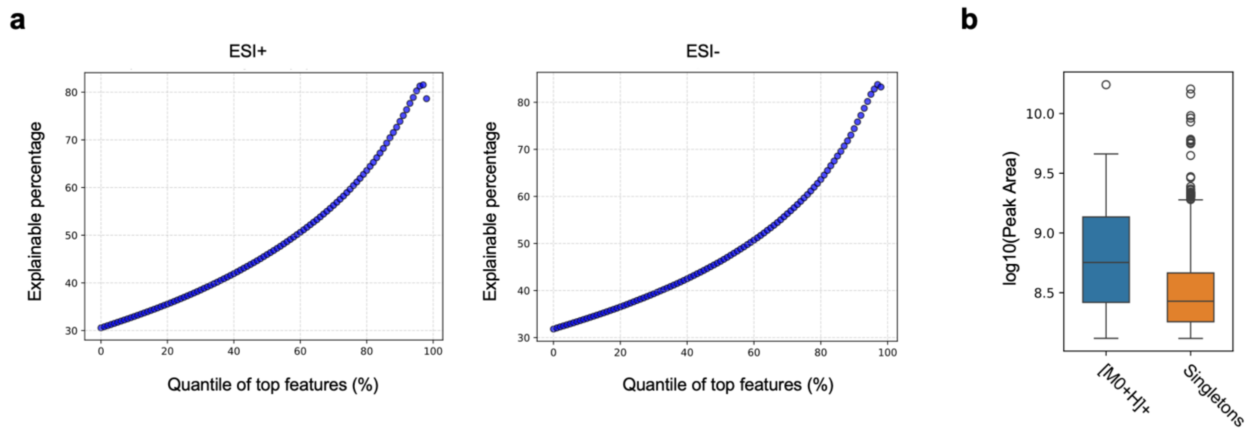

Supplement: Supplementary file 1 [file NIHPP2025.02.04.636472v1-supplement-2.pdf]
